# Quantitative spatial mapping of tissue water and lipid content using spatial frequency domain imaging in the 900- to 1000-nm wavelength region

**DOI:** 10.1117/1.JBO.27.10.105005

**Published:** 2022-10-27

**Authors:** Bowen Song, Xinman Yin, Yubo Fan, Yanyu Zhao

**Affiliations:** Beihang University, School of Engineering Medicine, Beijing Advanced Innovation Center for Biomedical Engineering, Key Laboratory for Biomechanics and Mechanobiology of Ministry of Education, Beijing, China

**Keywords:** water, lipid, spatial frequency domain imaging, tissue optics

## Abstract

**Significance:**

Water and lipid are key participants of many biological processes, but there are few label-free, non-contact optical methods that can spatially map these components *in-vivo*. Shortwave infrared meso-patterned imaging (SWIR-MPI) is an emerging technique that successfully addresses this need. However, it requires a dedicated SWIR camera to probe the 900- to 1300-nm wavelength region, which hinders practical translation of the technology.

**Aim:**

Compared with SWIR-MPI, we aim to develop a new technique that can dramatically reduce the cost in detector while maintaining high accuracy for the quantification of tissue water and lipid content.

**Approach:**

By utilizing water and lipid absorption features in the 900- to 1000-nm wavelength region as well as optimal wavelength and spatial frequency combinations, we develop a new imaging technique based on spatial frequency domain imaging to quantitatively map tissue water and lipid content using a regular silicon-based camera.

**Results:**

The proposed method is validated with a phantom study, which shows average error of 0.9±1.2% for water content estimation, and −0.4±0.7% for lipid content estimation, respectively. The proposed method is also demonstrated for *ex vivo* porcine tissue lipid mapping as well as *in-vivo* longitudinal water content monitoring.

**Conclusions:**

The proposed technique enables spatial mapping of tissue water and lipid content with the cost in detector reduced by two orders of magnitude compared with SWIR-MPI while maintaining high accuracy. The experimental results highlight the potential of this technique for substantial impact in both scientific and industrial applications.

## 1 Introduction

Water and lipid are key components of many biological processes and are inextricably linked to proper cellular function. Dynamics in the concentrations and spatial distribution of those components are hallmarks of many conditions including cardiovascular disease, inflammation, diabetes, and several cancers.^1^^–^^3^ In addition, quantitative and spatial information of those components have shown to be valuable for clinical applications such as tumor boundary detection in surgeries, dehydration assessment in sports medicine, and evaluation of skin aging.^4^^–^^7^

Visible (VIS: 400 to 700 nm) and near-infrared (NIR: 700 to 900 nm) light has long been used for quantification of tissue components such as oxy- and deoxy-hemoglobins in a label-free and non-invasive manner.^8^^–^^11^ For example, spatial frequency domain imaging (SFDI) is an optical technique that can quantify tissue hemoglobin concentrations and has been widely used for small animal monitoring, burn imaging, surgical guidance, and neuroscience.^12^^–^^15^ However, quantitative measurements for water and lipid are intrinsically challenging in the VIS and NIR wavelengths due to the absence of corresponding absorption features.^16^ In contrast, water and lipid possess distinct absorption characteristics in the shortwave infrared wavelengths (SWIR: 900 to 2500 nm),^10^^,^^11^ suggesting that use of this spectral region may enhance the accessibility of these species. For example, shortwave infrared meso-patterned imaging (SWIR-MPI) is an emerging variation on SFDI that utilizes the SWIR wavelengths to quantify water and lipid concentrations in a label-free, widefield manner.^17^

While commonly available silicon-based detectors are sensitive to wavelengths below 1100 nm, SWIR-MPI involves measurements covering the 900- to 1300-nm region, which dictates the use of SWIR detectors such as InGaAs or germanium-based cameras.^11^^,^^17^ However, the cost of those SWIR cameras is typically two orders of magnitude higher than silicon-based cameras, which hinders immediate practical applications of the technology.^18^^–^^21^ To address this bottleneck, here, we develop an optical technique based on SFDI that utilizes commonly available silicon-based detector, for quantitative, label-free optical imaging of tissue water and lipid content.

In this work, we first demonstrate the feasibility of quantifying water and lipid content with the 900- to 1000-nm wavelength region that is accessible by silicon-based detectors. We then conduct comprehensive numerical simulations to identify the optimal measurement wavelengths and spatial frequencies. We further validate the quantification of water and lipid content with a phantom study, and demonstrate potential applications of the proposed method with *ex-vivo* lipid content mapping as well as *in-vivo* longitudinal water content monitoring. Finally, we conclude with a discussion of pre-clinical, clinical, and industrial application areas in which the proposed technique may have a substantial impact.

## 2 Methods

### 2.1 Absorption Features of Water and Lipid in the 900- to 1000-nm Wavelength Region

**Fig. 1:**
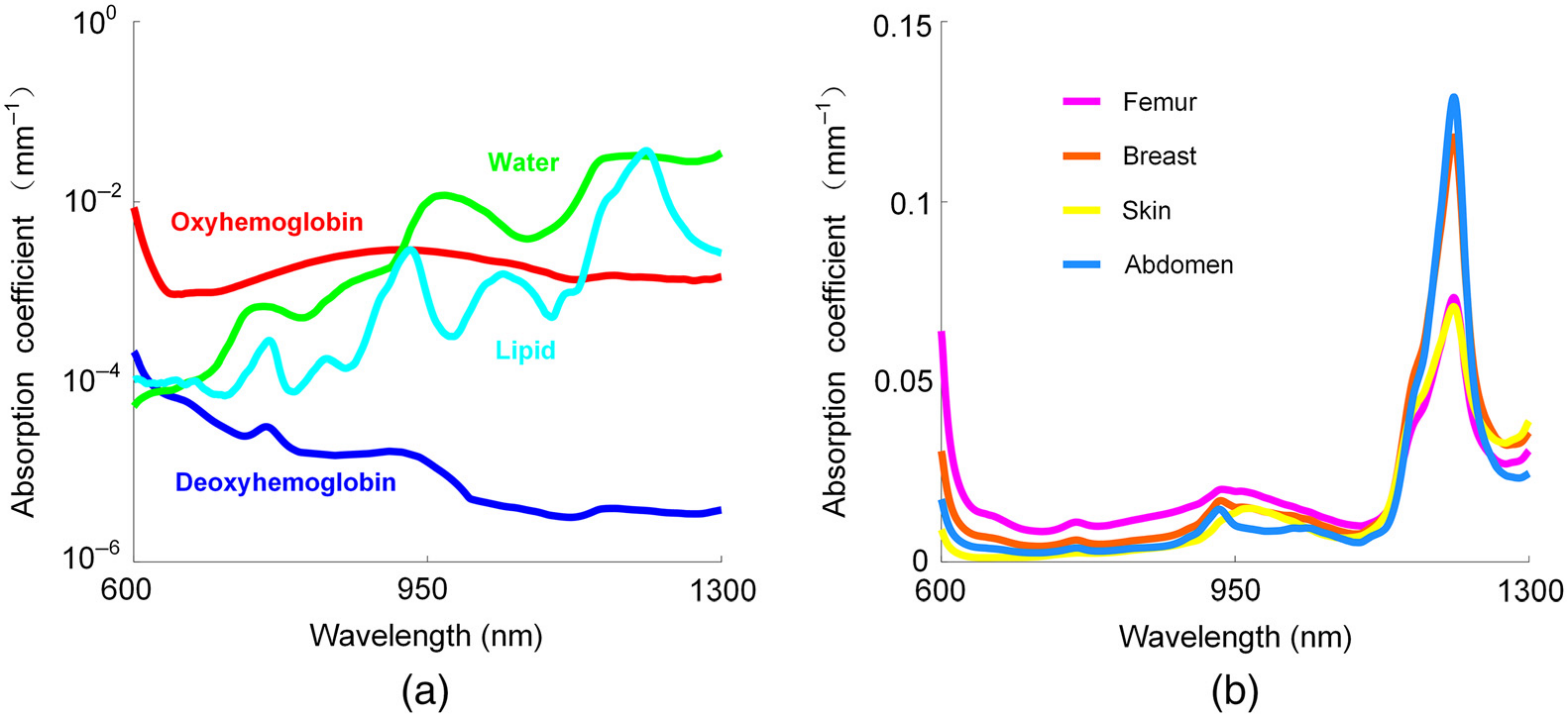
(a) Absorption spectra of oxyhemoglobin (9.5  μM), deoxyhemoglobin (0.08  μM), water (26.1%), and lipid (22.5%) contents of human skin in the 600- to 1300-nm wavelengths. (b) Absorption spectra in the 600- to 1300-nm wavelength range for various tissue types.

Figure 1 shows the absorption spectra of various chromophores as well as tissue types in the 600- to 1300-nm wavelength range.^22^^–^^26^ The quantitative mapping of tissue water and lipid content with structured light was initially demonstrated by SWIR-MPI that utilized the 900- to 1300-nm wavelength region.^17^ Specifically, SWIR-MPI primarily utilized the water absorption feature at 970 nm and the lipid absorption feature at 1210 nm. While the commonly used silicon-based cameras have access to the 970-nm wavelength, the 1210-nm wavelength is beyond their detection range, and requires specialized detectors such as InGaAs or germanium-based cameras. In other words, the lack of sensitivity to the lipid absorption feature near 1210-nm wavelength excludes the use of silicon-based detectors in SWIR-MPI. On the other hand, as shown in Fig. 1(a), although much smaller than the 1210-nm peak, there is another lipid absorption peak around 930 nm, which is within the sensitivity range of silicon-based detectors.^23^^,^^24^^,^^27^ In fact, fiber-based techniques such as diffuse optical spectroscopic imaging (DOSI) have been utilizing the 930- and 970-nm wavelengths to quantify tissue water and lipid content.^28^ Therefore, in this work, we explore the 900- to 1000-nm wavelength region and develop a hyperspectral SFDI system for the quantification of tissue water and lipid content. In addition, we note that SWIR-MPI is essentially SFDI operating in the SWIR wavelength region. They both use structured illumination to probe tissue optical properties (i.e., absorption and reduced scattering). The major difference is that SWIR-MPI operates in the SWIR region, whereas SFDI typically operates in the visible and near-infrared region. Consequently, in terms of detection hardware, SWIR-MPI requires InGaAs or germanium-based cameras, while typical SFDI systems use silicon-based cameras.

### 2.2 Spatial Frequency Domain Imaging

The details of SFDI image acquisition and processing have been described in detail elsewhere.^29^ Briefly, during image acquisition, SFDI projects spatially modulated light patterns onto the sample, and the reflectance images are collected by a camera. Typically, the spatial frequencies of the projected patterns (e.g., sinusoidal patterns projected along one-dimension) are carefully selected to ensure measurement accuracy.^30^ In data processing, the collected images are demodulated with Eq. (1), where $I_{1}$, $I_{2}$, and $I_{3}$ represent raw images acquired at each phase of the projected patterns.^31^ The diffuse reflectance image of spatial frequency $f_{x}$ is obtained by calibrating the demodulated images, as shown in Eq. (2), where $R_{d}(f_{x})$, $R_{d,\mathrm{ref}}(f_{x})$, $I(f_{x})$, and $I_{\mathrm{ref}}(f_{x})$ represent diffuse reflectance of the sample, diffuse reflectance of the reference phantom, demodulated image of the sample, and demodulated image of the reference phantom, respectively. The $R_{d}$ maps are then fed into a pre-computed Monte Carlo look-up-table (LUT) to extract optical absorption ($\mu_{a}$) and reduced scattering ($\mu_{s^{\prime }}$) coefficients in a pixel-by-pixel manner for each measurement wavelength. The chromophore concentrations, such as oxyhemoglobin, deoxyhemoglobin, water and lipid concentrations, can be extracted by fitting the measured absorption spectra using Beer’s law^32^^,^^33^

$$I=\frac{\sqrt{2}}{3}\sqrt{{\left(I_{1}-I_{2}\right)}^{2}+{\left(I_{2}-I_{3}\right)}^{2}+{\left(I_{3}-I_{1}\right)}^{2},}$$ (1)

$$R_{d}(f_{x})=\frac{I(f_{x})}{I_{\mathrm{ref}}(f_{x})}\times R_{d,\mathrm{ref}}(f_{x}).$$ (2)

### 2.3 Selection of Measurement Wavelengths and Spatial Frequencies

To identify optimal spatial frequencies for the measurement of water and lipid content with the 900- to 1000-nm wavelength region, we used the established Cramér–Rao bound (CRB) to explore SFDI measurement uncertainties as a function of wavelength under different spatial-frequency combinations.^30^ The noise parameters were obtained for our SFDI instrument following the procedures described in Pera et al.^30^ Specifically, the noise parameters were obtained with a set of 12 phantoms covering a wide range of optical properties in the 900- to 1000-nm wavelength range (i.e., 0.0067 to $0.082\;{\mathrm{mm}}^{-1}$ for absorption and 0.67 to $4.2\;{\mathrm{mm}}^{-1}$ for reduced scattering). The phantoms were homogeneous liquid phantoms with nigrosin and intralipid added water to control $\mu_{a}$ and $\mu_{s^{\prime }}$, respectively. Their optical properties spanned a range of values representative of the applications in our study, e.g., intralipid phantoms, porcine tissue, and small animal (mouse) imaging. In addition, a series of spatial frequency combinations were explored, including [0, 0.05], [0, 0.1], [0, 0.2], and $[0,0.4]\;{\mathrm{mm}}^{-1}$. It is worthy to note that the zero spatial frequency is constant in the pair because it helps accurate extraction of optical properties. As demonstrated in Pera et al.,^30^ using only a pair of higher spatial frequencies would yield inaccurate optical property estimations.

In addition, the choice of wavelengths is also critical for the measurement of water and lipid content in tissue. With the wavelength range of 900 to 1000 nm, one could conduct SFDI measurements with 1-nm increment, but it would take a long time for data acquisition and potentially cause motion artifacts for *in-vivo* measurements. Alternatively, one could also measure with 10 nm increments, but the small number of wavelengths might lead to large measurement errors. To investigate the tradeoff between the number of measurement wavelengths and the measurement accuracy for water and lipid content, we further conducted simulations for the concentration extraction with reference to physiological values in literature.^34^^–^^36^ Specifically, instead of choosing fixed increments, the concentrations of water and lipid were set to 15% to 90% and 20% to 80%, respectively, with reference to physiological values in literature.^34^^–^^36^ A total of 100,000 concentration combinations were randomly selected (using the rand function in MATLAB) from the above ranges, and corresponding absorption spectra were generated. In addition, zero-mean Gaussian noise was added to the absorption values of each wavelength increment, where the standard deviation was determined by the CRB measurement uncertainty.^30^ The water and lipid concentrations were then calculated using Beer’s law with the absorption spectra, which ranged from 900 to 1000 nm with increments of 1, 2, 5, and 10 nm, respectively. For each wavelength increment, the average and standard deviation of percent error were calculated by comparing the percent difference between the extracted concentrations from the generated absorption spectra and the known ground truth values. Since the generated absorption spectra have zero-mean Gaussian noise, the average percent errors of each wavelength increment would be close to zero, and the standard deviation of the percent errors would indicate the goodness of the concentration extraction. In addition, oxyhemoglobin and deoxyhemoglobin were not included for the wavelength selection because (1) the simulation study was intended to identify wavelengths for accurate water and lipid content estimation and (2) the estimation of oxyhemoglobin and deoxyhemoglobin has been extensively studied by Mazhar et al.^32^ and requires a wavelength region below 900 nm for accurate extraction.

### 2.4 Optical Instrumentation

**Fig. 2:**
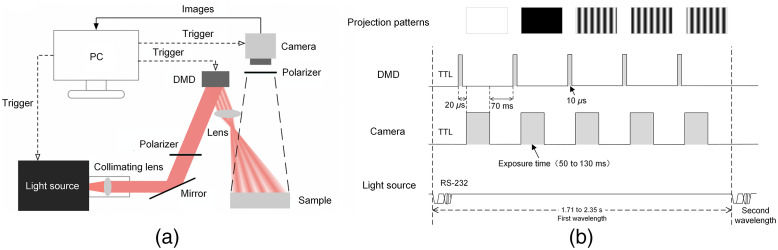
(a) SFDI system diagram. (b) Synchronization sequence of the system components.

The SFDI system built for experimental validation of this study is shown in Fig. 2(a). The light source is composed of a tungsten halogen lamp with a wavelength range of 300 to 2500 nm (HL1000, NBeT, Beijing, China) and a monochromator (HGISW151, HGOAN, Beijing, China). The spectral resolution (i.e., FWHM) of the monochromator (with fully opened slits to maximize light throughput) is ∼1 to 2 nm according to the manufacturer. The output from the light source is collimated and sent to a digital micromirror device (DMD, V-650L, ViALUX, Saxony, Germany) for spatial modulation. A series of spatially modulated light patterns (shifted 120 deg sequentially in phase) are then projected onto the sample, and the reflectance images are collected by a silicon-based camera (BFS-U3-04S2M-CS, FLIR, Oregon). In addition, five projection patterns were used for each wavelength. Specifically, a “white” and a “black” pattern were used for the $0\;{\mathrm{mm}}^{-1}$ spatial frequency, and three sinusoidal patterns (shifted 120-deg sequentially in phase) were used for the $0.2\text{-}{\mathrm{mm}}^{-1}$ spatial frequency. The camera exposure time in our study was 50 to 130 ms. Correspondingly, the measurements took 1.71 to 2.35 s per wavelength. An achromatic lens with 75-mm focal length and 50.8-mm diameter (GLH-31, Heng Yang Guang Xue, Shenzhen, China) was used as the projection lens. Orthogonal linear polarizers (#12-474, Edmund Optics, New Jersey) are used to reduce specular reflection from the sample surface. The image acquisition of the camera, spatial modulation of the DMD, and the output wavelength of the light source are synchronized and controlled by a custom software on the personal computer (PC). The synchronization sequence of the system components is shown in Fig. 2(b). The light source is first set to the desired wavelength by the control software through serial communication (i.e., RS-232). Then, the DMD is triggered with a 10-μs transistor-transistor logic (TTL) signal to project illumination patterns with specific spatial frequency and phase. The camera subsequently captures the reflectance images upon another TTL signal that starts 20  μs after the TTL for DMD. The camera exposure time is 50 to 130 ms in this study depending on different measurement samples. After exposure, the control program waits 70 ms for the acquired image to be transferred to the host computer and saved on hard drive. Subsequently, another 10-μs TTL signal is sent to the DMD to trigger the next projection pattern. The duration of one pattern projection is therefore ∼70  ms plus camera exposure time. The acquisition process is repeated for each measurement wavelength. The time between wavelengths is 1.71 to 2.35 s depending on different camera exposure times. The TTL signals are generated by an Arduino board controlled by the custom software, which is publicly available for download.^37^ In addition, the system is mounted on a 65×40  cm aluminum plate, and the physical size of the entire system is approximately within 80×40×40  cm. The imaging field-of-view of the system is 3.2×2.1  cm. Furthermore, a drift test was conducted over 2 h to demonstrate measurement robustness (Note S1 in the Supplementary Material). For data processing, the computation was conducted using a desktop computer with Intel i9-9900K CPU and 64 GB RAM. The inversion from raw data to optical properties took 0.98 s per wavelength, and the extraction of chromophore maps from optical property maps took 0.76 s.

### 2.5 Experimental Validations

For experimental validations, we first conducted a phantom study to validate the quantification of water and lipid content with the proposed method. We then conducted lipid content mapping with *ex-vivo* porcine tissue, as well as *in-vivo* longitudinal water content monitoring with small animals.

Liquid phantoms with specific proportions of water and lipid were made by varying ratios of water and intralipid (Fresenius Kabi SSPC, Jiangsu, China). A total of five phantoms were made for each concentration. The SFDI measurements were conducted on liquid phantoms ranging from 5% to 20% lipid with 900 to 1000 nm wavelengths of 5-nm increments and $[0,0.2]\;{\mathrm{mm}}^{-1}$ spatial frequencies. With reference to Tabassum et al.,^12^ a small 10% intralipid phantom was placed in the field-of-view for measurement correction. The water and lipid concentrations were estimated by fitting the measured absorption spectra using Beer’s law and then compared with known values. For each concentration, the average and standard deviation of error was calculated by comparing the difference between the extracted concentrations and the known values. The extracted water and lipid concentrations of a single phantom were calculated over an ∼1×0.5  cm region-of-interest at the center of the 2×1  cm phantom surface. In addition, the calibration phantom used in this study was 10% intralipid whose optical properties have been characterized in the literature.^23^^,^^24^ Specifically, to compute the extracted concentration values and errors, for a given water-lipid recipe, spatially averaged water and lipid concentrations were computed for each phantom, and then the average and standard deviation of those spatially averaged concentrations across the five phantoms for a given recipe were computed and listed under “extracted concentrations” in Table 1 (and Table S2 in the Supplementary Material). In addition, for every pixel in every phantom, the difference between the pixel value and the known concentration was computed. Then, the computed differences for each phantom were spatially averaged. For a given water-lipid recipe, the average and standard deviation of those spatially averaged differences were computed across the five phantoms, and those values were listed under errors in Table 1 (and Table S2 in the Supplementary Material).

**Table 1:** Extracted water and lipid concentrations. Five phantoms were measured per water-lipid recipe.

Known concentrations		Extracted concentrations		Errors	
Water (%)	Lipid (%)	Water	Lipid	Water	Lipid
95	5	95.8 ± 0.3%	4.7 ± 1.0%	0.8 ± 0.3%	−0.3 ± 1.0%
90	10	89.8 ± 0.2%	9.7 ± 0.3%	−0.2 ± 0.2%	−0.3 ± 0.3%
85	15	85.3 ± 0.4%	14.6 ± 0.7%	0.3 ± 0.4%	−0.4 ± 0.7%
80	20	82.8 ± 0.2%	19.2 ± 0.5%	2.8 ± 0.2%	−0.8 ± 0.5%

In addition, while lipid content has been an important parameter in the evaluation of meat product,^38^^,^^39^ we demonstrated the proposed method on lipid content mapping for an *ex vivo* porcine tissue purchased from a local supermarket. The measurement was conducted using $[0,0.2]\;{\mathrm{mm}}^{-1}$ spatial frequencies and 900- to 1000-nm wavelengths with 5-nm increments. Additionally, oxyhemoglobin and deoxyhemoglobin were not extracted for the *ex vivo* porcine tissue with reference to recent literature.^40^ Specifically, Lam et al. utilized narrowband hyperspectral diffuse reflectance in the 900- to 1000-nm range to get point estimations on tissue water and lipid content, in which oxyhemoglobin and deoxyhemoglobin were not extracted potentially due to the absence of blood content in the *ex vivo* tissues.

The accumulation of tissue water content in the extravascular interstitial space (i.e., edema) is closely associated with many physiological processes, such as tissue healing, inflammation, and sports injury.^41^ Therefore, we further demonstrated the proposed method for the monitoring of the presence, extent, and time course of *in-vivo* tissue water content. Specifically, tissue edema was simulated using subcutaneous injections of phosphate-buffered saline (PBS; no injection, 0.1 ml, or 0.2 ml) in the flank of BALB/c mice. A total of four mice were included for each injection group. With regard to “no injection,” we note that it would also be good to still insert the needle but not inject anything to control for the effect of inserting the needle for the actual PBS injections. The measurements were conducted using $[0,0.2]\;{\mathrm{mm}}^{-1}$ spatial frequencies and 650- to 1000-nm wavelengths with 5-nm increments. The measurements were repeated every 5 min for a total of 20 time points. With reference to our prior work, a silicone phantom was placed in the field of view for the correction of measurements over time.^12^ Chromophore concentrations were extracted for water, lipid, oxyhemoglobin, and deoxyhemoglobin using Beer’s law. The water content of each mouse was calculated by spatially averaging the water content map with a 0.5-cm diameter around the injection site. The averages and standard deviations were then computed from the four water content values of each group. In addition, oxyhemoglobin and deoxyhemoglobin were included in the chromophore fitting since they are also major absorbers (in addition to water and lipid) for *in-vivo* tissues in the 900- to 1000-nm wavelength range. The 650- to 1000-nm wavelength range was used with reference to prior DOSI literature for the extraction of oxyhemoglobin, deoxyhemoglobin, water, and lipid.^34^^–^^36^ Additionally, the PBS injection was conducted immediately after three repeated baseline measurements, after which 17 measurements were made with a time interval of 5 min between measurements. The animals were under anesthesia during the procedure with ∼2% isoflurane anesthesia. All animal procedures were reviewed and approved by the Beihang University Biological and Medical Ethics Committee.

## 3 Results

### 3.1 Selection of Measurement Wavelengths and Spatial Frequencies

**Fig. 3:**
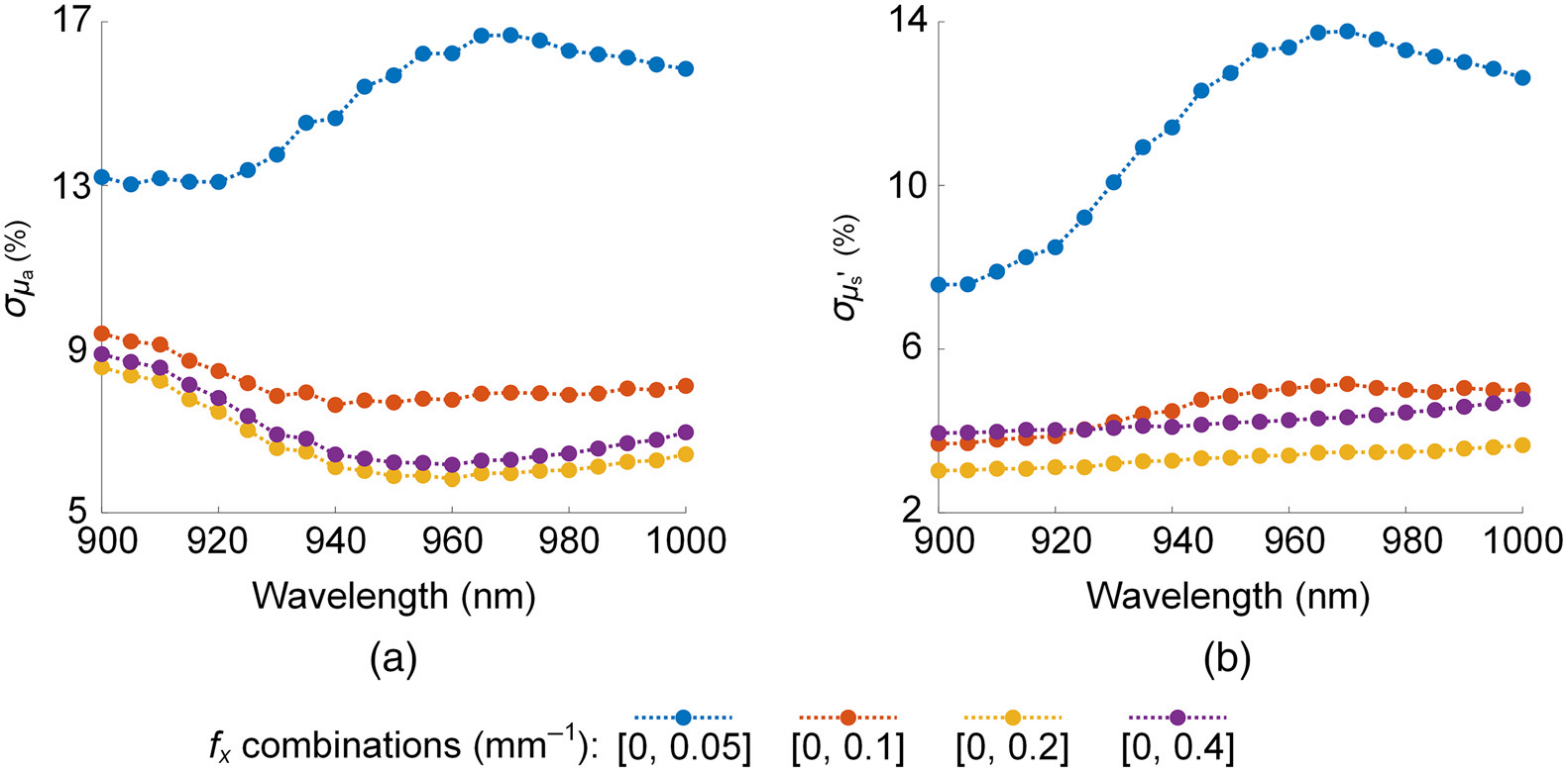
Measurement uncertainties of (a) absorption and (b) reduced scattering for different spatial frequency combinations in the 900- to 1000-nm wavelength region.

The measurement uncertainties in optical absorption and reduced scattering are shown in Fig. 3 for wavelengths ranging from 900 to 1000 nm with 5-nm increments. The results show that the spatial frequency combination of $[0,0.05]\;{\mathrm{mm}}^{-1}$ have the largest measurement uncertainties among the four combinations. In contrast, the $[0,0.2]\;{\mathrm{mm}}^{-1}$ and $[0,0.4]\;{\mathrm{mm}}^{-1}$ both have relatively low uncertainties. With reference to Cuccia et al.,^31^ we further calculated the effective penetration depth of the 0.2 and $0.4\;{\mathrm{mm}}^{-1}$ spatial frequencies using optical properties of tissue-mimicking 10% intralipid phantom. The average effective penetration depth of $0.2\;{\mathrm{mm}}^{-1}$ and $0.4\;{\mathrm{mm}}^{-1}$ over the 900- to 1000-nm wavelengths were 2 and 1.6 mm, respectively. With reference to Hayakawa et al.,^42^ we also calculated the optical sampling depth (i.e., photon 90th percentile depth penetration) of the 0.2 and $0.4\;{\mathrm{mm}}^{-1}$ spatial frequencies using optical properties of tissue-mimicking 10% intralipid phantom. The average photon 90th percentile depth penetration over the 900 to 1000 nm wavelengths was 0.93 mm for $0.2\;{\mathrm{mm}}^{-1}$ and 0.55 mm for $0.4\;{\mathrm{mm}}^{-1}$, respectively. To give more information, the optical sampling depth and effective depth penetration were plotted and shown in Fig. S2 in the Supplementary Material for 900- to 1300-nm wavelength range and $[0,0.05,0.1,0.2,0.4]\;{\mathrm{mm}}^{-1}$ spatial frequencies. Furthermore, it is noted that the 0 and $0.2\;{\mathrm{mm}}^{-1}$ spatial frequencies each probes a different depth of the tissue, which is known as the partial volume effect and represents a limitation of SFDI (and other diffuse optical imaging methods). The partial volume effect could potentially be minimized by using other spatial frequency pairs that are close to each other, such as $[0.05,0.1]\;{\mathrm{mm}}^{-1}$. However, this would lead to larger measurement uncertainties, which has been demonstrated in Pera et al.^30^ In addition, we have also calculated optical property measurement uncertainties using spatial frequency combinations such as [0.05, 0.1], [0.05, 0.2], [0.1, 0.2], and $[0.05,0.4]\;{\mathrm{mm}}^{-1}$ (Fig. S3 in the Supplementary Material). It shows that in comparison to the $[0,0.2]\;{\mathrm{mm}}^{-1}$ pair, other spatial frequency pairs without $0\;{\mathrm{mm}}^{-1}$ consistently have larger measurement uncertainties. Given the relatively lower measurement uncertainty as well as larger effective penetration depth and optical sampling depth, the spatial frequency combination of $[0,0.2]\;{\mathrm{mm}}^{-1}$ was selected for this study.

**Fig. 4:**
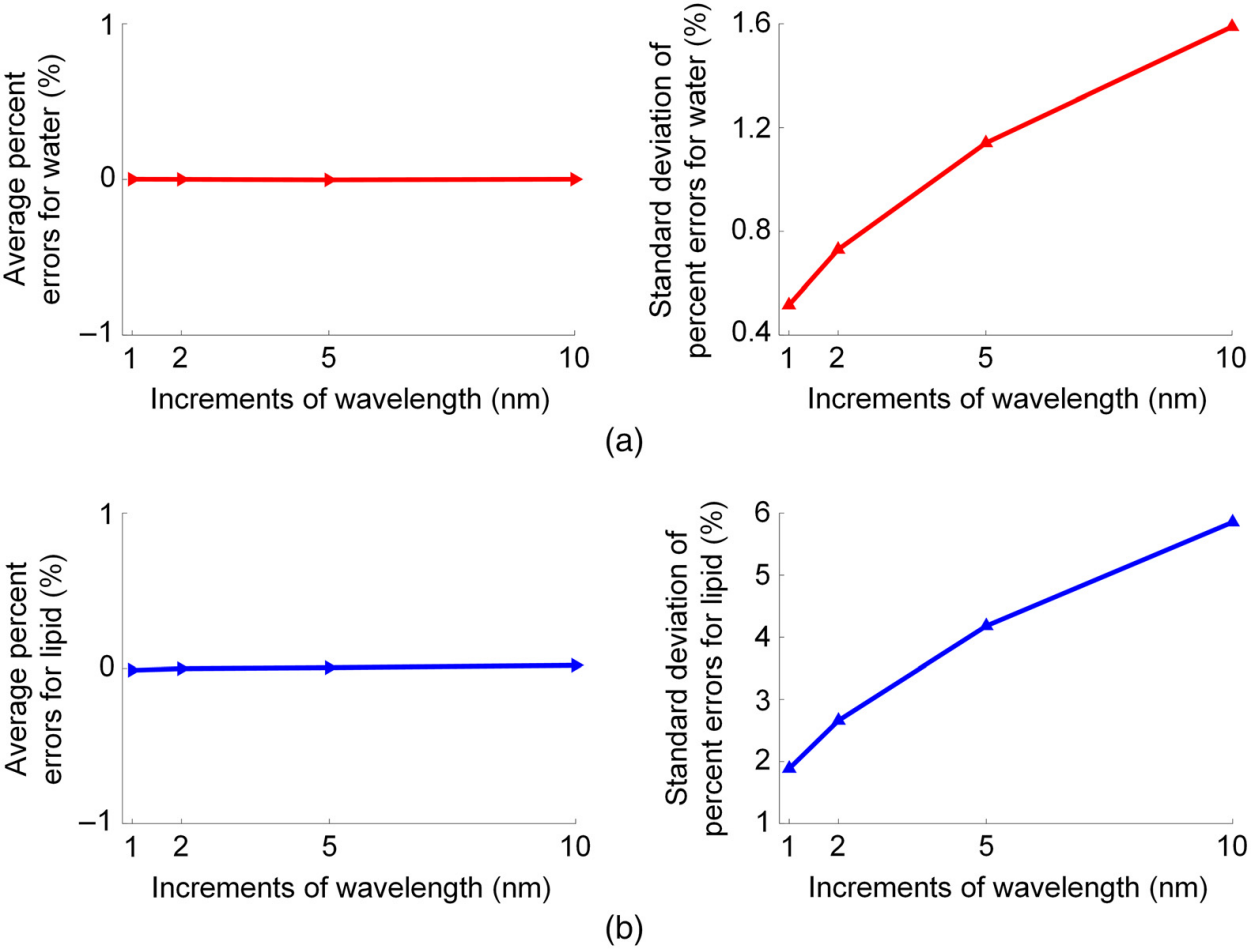
Average and standard deviation of percent errors of extracted concentrations for (a) water and (b) lipid. It is worthy to note that since the magnitude of average percent errors were close to zero (all below 0.02% for both water and lipid) for different wavelength increments under zero-mean Gaussian noise, the standard deviations were used to assess the goodness of concentration extraction.

The average and standard deviation of percent errors of the extracted water and lipid concentrations are shown in Fig. 4 for each wavelength increment. It is worthy to note that since the magnitude of average percent errors were close to zero (all below 0.02% for both water and lipid) for different wavelength increments under zero-mean Gaussian noise, the standard deviations were used to assess the goodness of concentration extraction. As expected, the 10-nm increment (i.e., a total of 11 measurement wavelengths in the range of 900 to 1000 nm) led to the largest errors out of the four assessed wavelength increments. The smallest errors were achieved with 1-nm increment (i.e., a total of 101 measurement wavelengths). Overall, the measurement errors decrease with decreasing increment of wavelength, but the required data volume and time cost increase accordingly. Therefore, the 5-nm increment was selected in this study as a tradeoff between accuracy and increment of measurement wavelength.

### 3.2 Phantom Study for the Validation of Water and Lipid Content Quantification

A series of homogeneous phantoms with varying water and lipid content was measured with the proposed method to validate the accuracy of chromophore extraction. The average error for water content estimation calculated over all phantoms was 0.9±1.2%, and the average error for lipid was −0.4±0.7% over a wide physiological range (5% to 20% for lipid, 80% to 95% for water).^22^^,^^36^^,^^38^^,^^43^ In addition, for each concentration combination, the average and standard deviation of extracted water and lipid content are given in Table 1 and compared with the known concentrations (with one significant digit after the decimal). For comparison, given intralipid phantoms of the same water and lipid concentrations in the SWIR-MPI work,^17^ the average error for water content estimation was −0.2±2.5%, and the average error for lipid was 0.3±1.6%. While the amplitudes of the average errors for water and lipid were larger with the presented method, the standard deviations were smaller than those of the SWIR-MPI, suggesting comparable accuracies between the two methods. These results demonstrate that the proposed method can extract water and lipid concentrations with high accuracy. In addition, representative water and lipid concentration maps for each water-lipid recipe are shown in Fig. S4 in the Supplementary Material.

### 3.3 Lipid Content Mapping on *Ex-Vivo* Porcine Tissue

**Fig. 5:**
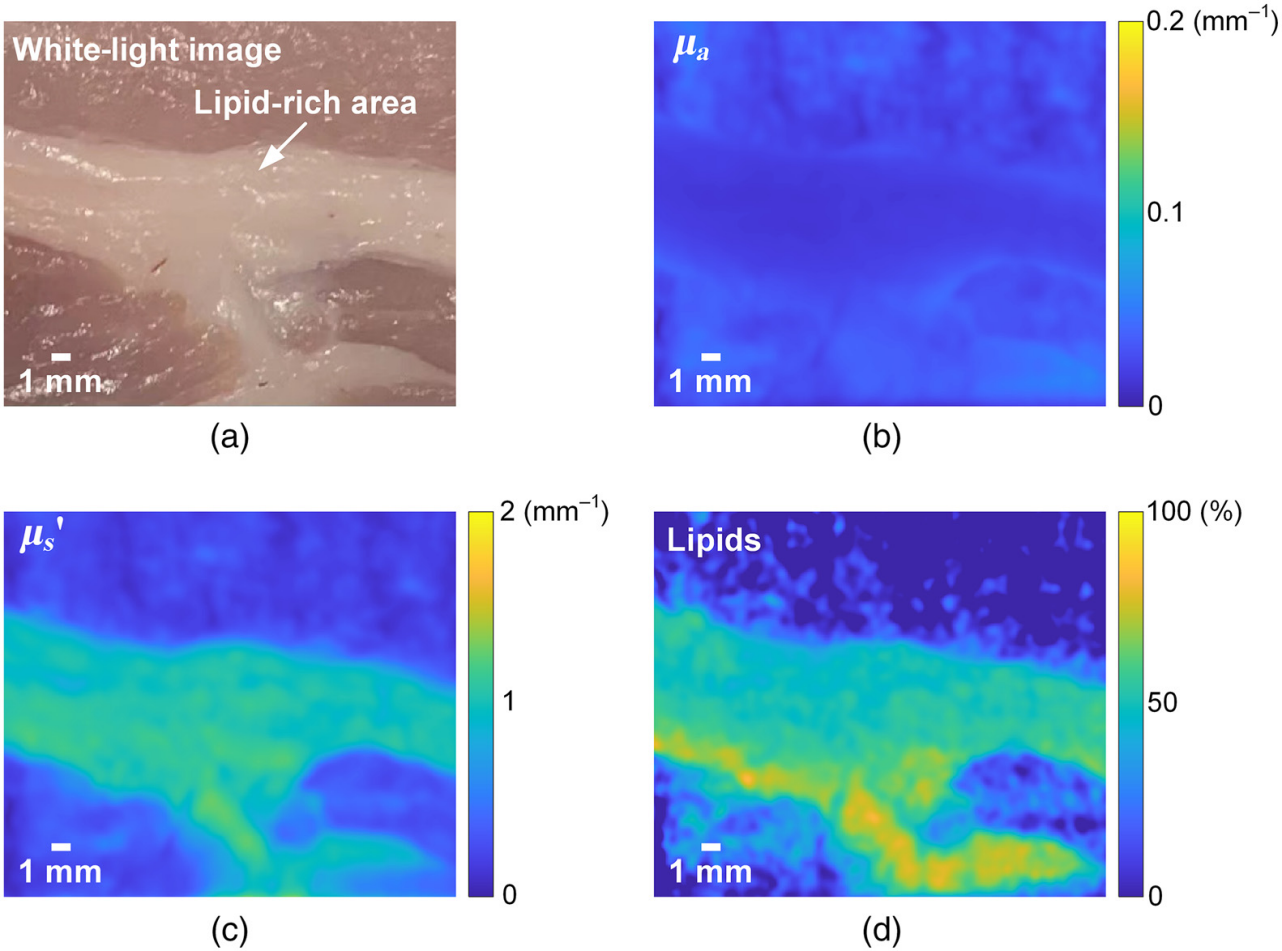
(a) White-light image of the porcine tissue. (b) Absorption map at 930 nm. (c) Reduced scattering map at 930 nm. (d) Extracted lipid content map of the porcine tissue. No pixels in the maps are not a number (NaNs).

Lipid content has been an important parameter in meat product grading and evaluation.^38^^,^^39^ We demonstrate lipid content mapping of an *ex vivo* porcine tissue purchased from a local supermarket. The white-light image of the tissue is shown in Fig. 5(a), where the lipid-rich area is visually apparent. The absorption and reduced scattering maps at 930 nm are shown in Figs. 5(b) and 5(c), respectively. The lipid content map is shown in Fig. 5(d), where the lipid concentration is quantified and spatially mapped. The results demonstrate that the proposed method can potentially be applied to widefield quantitative evaluation of meat products such as porcine tissues.

### 3.4 *In-Vivo* Water Content Monitoring on Small Animals

**Fig. 6:**
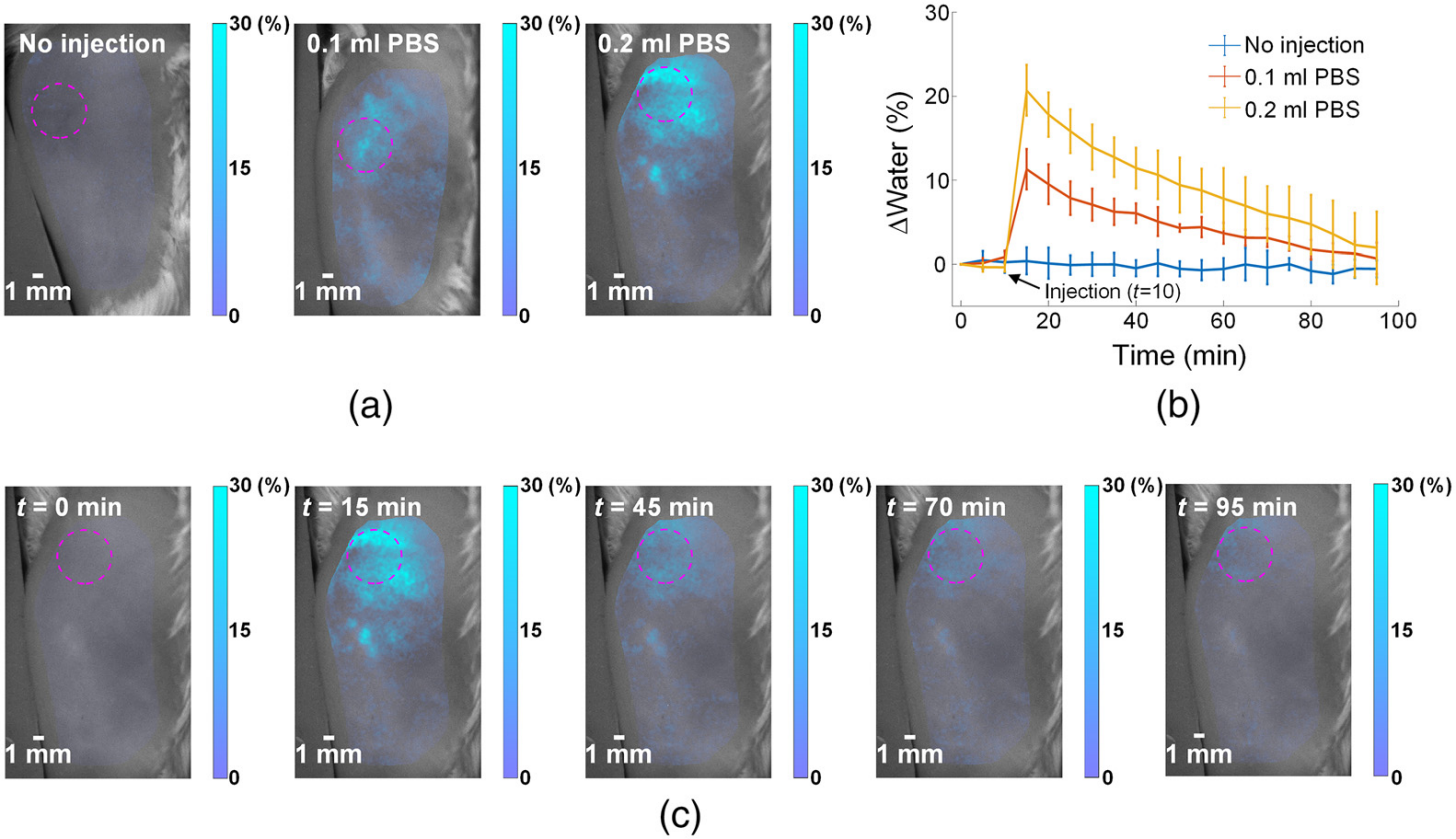
Longitudinal monitoring of *in-vivo* water content. (a) Representative mice are shown for the changes of water content immediately after subcutaneous injection of PBS (n=4 per group). The magenta dashed circle is centered at the injection site and represents the 0.5-cm diameter region-of-interest used for calculating the average water content. (b) Time series of average water content changes for each group (error bars indicate standard deviations). (c) Temporal dynamics of water content on a representative mouse in the 0.2-ml group.

Tissue edema is closely associated with many physiological processes such as tissue healing, inflammation, and sports injury.^41^ The tissue edema was simulated by no injection as control group, and subcutaneous injections of 0.1 or 0.2 ml PBS, respectively on three groups of mice (n=4). Figure 6(a) shows the spatial distribution and magnitude of water content changes immediately after injection, where a dose-dependent effect is clearly demonstrated. Figure 6(b) shows the time series of the changes in average water content. The average water content was calculated by spatially averaging the water content map with a 0.5-cm diameter circular region-of-interest centered at the injection site [shown as magenta dashed circles in Figs. 6(a) and 6(c)]. The injections were conducted immediately after three repeated baseline measurements. Figure 6(c) shows the temporal dynamics of the water content changes on a representative mouse with 0.2-ml PBS injection. These results demonstrate that the proposed method can longitudinally track water content for *in-vivo* applications.

## 4 Discussion

In this work, we have developed and validated SFDI in the 900- to 1000-nm wavelength region for label-free, non-contact mapping of tissue water and lipid content. We conducted comprehensive numerical simulations to identify optimal spatial frequency and wavelength combinations for the quantification of water and lipid content with the 900- to 1000-nm region. We then validated the extraction of water and lipid content in a phantom study using the identified spatial frequencies and wavelengths. We further demonstrated the technique for *ex vivo* lipid mapping in porcine tissue, and *in-vivo* longitudinal water content monitoring in small animal model, indicating potential applications in food industry and small animal studies. Additionally, we provide comparisons of the presented method with SWIR-MPI and a discrete wavelength version (e.g., three wavelengths) of the presented work (Note S2 in the Supplementary Material).

In addition to the SWIR-MPI work,^17^ Wilson et al. for the first time extended SFDI from NIR into the SWIR region up to 1800 nm with a hybrid illumination scheme using an InGaAs camera,^44^ which could also have been used for the quantification of tissue water and lipid content. To the best of our knowledge, our presented method is the first demonstration of quantifying and mapping water and lipid concentrations with SFDI using a silicon-based detector.

This technique enjoys a number of advantages over current methods used for quantifying tissue water and lipid content. For example, fluorescence imaging can map tissue lipid content, but requires exogenous agents and does not simultaneously map water content.^45^ Magnetic resonance imaging (MRI) can generate tomographic volumes of water and lipid contrast with T1 and T2 weighted scans, but has much higher cost and is impractical for routine monitoring in the primary care setting.^46^ MRI can also image much larger area and significantly deeper, albeit with lower spatial resolution. Compared with DOSI which can measure deeper and map water and lipid with fiber-based probe, SFDI is inherently non-contact and widefield, and has significantly higher spatial resolution (i.e., sub-mm).^47^ In terms of temporal resolution, recent halftone-SFDI technology has demonstrated SFDI measurements with kilohertz speed, which is orders of magnitude faster than typical DOSI measurements.^48^ Another fiber-based technique was proposed by Lam et al.^40^ to quantify water and lipid content using calibrated reflectance in the 900- to 1000-nm wavelength range without temporal or spatial modulation of light. Specifically, reduced scattering was assumed as a constant value over the entire wavelength region. Water and lipid contents were assumed to be the only absorbers in the turbid media and then quantified by fitting the estimated absorption spectra. While absolving the need for scattering quantification, it would be challenging to apply this fiber-based technique for *in-vivo* tissues where oxyhemoglobin and deoxyhemoglobin are also major absorbers in addition to water and lipid. In contrast, our proposed method can quantify both absorption and reduced scattering over a wide wavelength range, and can be applied to *in-vivo* tissues. Furthermore, compared with SWIR-MPI that requires wavelengths up to 1300 nm and a dedicated SWIR camera, the presented method takes advantage of the 900- to 1000-nm region and utilizes a regular silicon-based camera whose cost is typically two orders of magnitude lower than SWIR cameras.^18^^–^^21^ In addition, compared with the SWIR-MPI system reported in previous literature,^17^ the proposed low-cost system has additional advantages in terms of compactness, portability, and speed. Specifically, the light source in the proposed system is a tungsten halogen lamp coupled to a monochromator, which is compact and portable. In comparison, the previous SWIR-MPI system utilized a tunable femtosecond laser which had to be fixed on optical table. Additionally, the system was mounted on a 65×40  cm aluminum plate, and the physical size of the entire system was ∼80×40×40  cm, making the proposed system suitable for *in-vivo* and clinical measurements. Furthermore, while the SWIR-MPI system required 30 s per wavelength for acquisition, the presented system only used 1.71 to 2.35 s per wavelength, which is 12.8 to 17.5× faster, demonstrating a speed improvement over an order of magnitude.

The proposed work has some limitations of note. For example, the lipid absorption at the 930 nm wavelength is lower than that of the 1210 nm utilized in the SWIR-MPI, which may lead to decreased sensitivity to lipid content. This could be overcome by sampling the wavelength region with relatively small increments such as 5 nm as demonstrated in the lipid titration experiment. Additionally, a small 10% intralipid phantom was used in this study (while not used in the case of SWIR-MPI) for measurement correction.^12^^,^^49^ This was because the output of the tungsten halogen lamp had drifts over timescale of hours, which would cause larger measurement errors. In contrast, for the porcine imaging, the small phantom for measurement correction was not used since the tissue measurement was conducted immediately after calibration. While the use of small phantom for correction can improve measurement accuracy, it would lead to reduced field-of-view of the system. To overcome this limitation, one could potentially utilize hardware with higher temporal stability to absolve the use of the correction phantom. In addition, the field-of-view in the presented work is limited by the output optical power of the light source (composed of a tungsten halogen lamp and a monochromator). Due to the small entrance slit size of the monochromator (i.e., millimeter scale) and the large bulb size of the tungsten halogen lamp (i.e., several centimeters), only a small portion of the light from the lamp could be coupled into the monochromator (using regular optical lenses), resulting in limited optical power at each output wavelength. A low-cost solution to this limitation could be changing the tungsten halogen lamp into a xenon lamp. While the spectral output of tungsten halogen lamps is smoother, the xenon and tungsten halogen lamps have comparable overall output intensity in the 900- to 1000-nm range. The xenon lamp typically has a bulb size on the sub-millimeter scale and can be regarded as a point source in practice. As a result, a much larger portion of the output light from the bulb would be able to enter the monochromator, which leads to increased illumination power and consequently allows for larger imaging field-of-view (i.e., achieving the same SNR with larger illumination area). Additionally, since the size of field-of-view is fundamentally limited by SNR, another solution to the limited field-of-view is to use detectors with lower noise. Detailed SNR analysis, comparison, and ways of improvement are provided in Note S3 in the Supplementary Material (our analysis suggests potential SNR improvement by ∼40 to 100× with cameras of significantly lower noise).

Going forward, the proposed technique can be applied to bedside or clinical monitoring of edema. While the skin aging is related to subcutaneous water content, the proposed technique can potentially be used to evaluate skin conditions as well as skincare products.^50^^,^^51^ In addition, it may also be a useful tool for meat product screening and grading.

In summary, this work introduced an SFDI technique that is able to quantitatively map tissue water and lipid content using a cost-effective silicon-based detector. This method may have substantial impact for scientific and industrial applications such as small animal monitoring and meat evaluation.
