# Supplementary material for: Quantitative spatial mapping of tissue water and lipid content using spatial frequency domain imaging in the 900- to 1000-nm wavelength region
Source: J Biomed Opt. 2022 Oct 27;27(10):105005. doi: 10.1117/1.JBO.27.10.105005 (PMC9612091; doi:10.1117/1.JBO.27.10.105005)
Supplement: Supplementary file 1 [file JBO_027_105005_SD001.pdf]

Supplementary Information

Spatial mapping of tissue water and lipid content using spatial frequency domain imaging in the 900-1000 nm wavelength region

Bowen Song,[a] Xinman Yin,[a] Yubo Fan,[a,*] Yanyu Zhao[a,*]

[a]Beijing Advanced Innovation Center for Biomedical Engineering, Key Laboratory for Biomechanics and Mechanobiology of Ministry of Education, School of Engineering Medicine, Beihang University, 100191, Beijing, China

***** Yubo Fan**,** E-mail: yubofan@buaa.edu.cn; Yanyu Zhao**,** E-mail: yanyuzhao@buaa.edu.cn

Supplementary Note 1 – Drift test

A drift test was conducted over a course of 2 hours. Specifically, 10% intralipid phantom was repeatedly measured during the 2 hours with 30 min interval, and water and lipid concentrations were extracted. The measurements were conducted using 900-1000 nm wavelengths with 5 nm increments and [0, 0.2] mm^{-1} spatial frequencies. With reference to our prior work,[12] a phantom with known optical properties was placed in the field-of-view for the correction of measurements over time. The extracted optical absorption and reduced scattering at different wavelengths are compared with expected values in Fig. S1 (a) and (b), respectively. The mean and standard deviations of the measured optical properties are plotted on top of the expected optical property spectra, which shows good agreement with ground truth values and small variation between measurements. The coefficient of variation (defined as the ratio of the standard deviation to the mean) is calculated for the measured optical absorption and reduced scattering, as shown in Fig. S1 (c) and (d), respectively. The average coefficient of variation at different wavelengths are 0.6±0.2% for absorption measurements and 0.9±0.1% for reduced scattering measurements. The extracted water and lipid concentrations during the drift test are compared with known

concentrations in Table S1, which also demonstrates good agreement with the ground truth, with an average error of 0.2±0.5% for water and 0.5±0.5% for lipid.

Supplementary Note 2 – Comparison with SWIR-MPI and a discrete wavelength version of the presented method

The proposed method presented results on phantom validation for the quantification of water and lipid contents, lipid mapping for *ex vivo* porcine tissue, and *in vivo* water content monitoring for small animals. Here we provide comparisons to results from both SWIR-MPI and a discrete wavelength version of the presented method. Additionally, we further compare the extraction of water and lipid content of the presented method with SWIR-MPI and the version of discrete wavelengths with comprehensive simulations.

1. Comparison with results from SWIR-MPI

In the phantom validation study, both SWIR-MPI and the presented method utilized liquid phantoms comprising four specific proportions of water and lipid (i.e., 5%, 10%, 15%, and 20% lipid content). With SWIR-MPI, the average error for water content estimation was -0.2 ± 2.5%, and the average error for lipid was 0.3 ± 1.6%. With the presented method, the average error for water content estimation was 0.9 ± 1.2%, and the average error for lipid was -0.4 ± 0.7%. The results show that the presented method has comparable accuracy for water and lipid content estimation compared to SWIR-MPI.

In the lipid mapping for porcine tissue, the estimated lipid content at lipid-rich area was approximately 40-50%. In contrast, in the SWIR-MPI work, the lipid content at lipid-rich area of the pork belly tissue was approximately 20-30%. Such discrepancy could be due to the partial volume effect, different probing depth between the measurement wavelength regions, and the fact

that the tissues being measured were two distinct pork samples. It would be interesting to measure the same porcine tissue with both SWIR-MPI and the presented method for comparison. Unfortunately, our lab (focusing on biomedical optics) at Beihang University is not yet able to conduct any SWIR-MPI measurements, since the purchase request for scientific-grade SWIR camera was denied by multiple vendors, probably due to the fact that the university is on the Entity List of the Export Administration Regulations (EAR) determined by the U.S. Government. We are currently working on purchasing the SWIR camera from other channels such as European vendors.

In the *in vivo* water monitoring study, both the presented method and SWIR-MPI were used to monitor three groups of mice (n=4 per group), with each group being injected 0 ml, 0.1 ml, and 0.2 ml PBS, respectively. With SWIR-MPI, the measured changes of water content upon immediate PBS injection were 12.9% for 0.1 ml and 20.1% for 0.2 ml, respectively. On the other hand, with the presented method, the measured changes of water content upon immediate PBS injection were 10.5% for 0.1 ml and 21.1% for 0.2 ml, respectively, which are close to the results by SWIR-MPI.

2. Comparison with results from a discrete wavelength version of the presented method

We followed the methods described in Mazhar et al.,[32] and conducted condition number analysis to select 3 discrete wavelengths in the 900-1000 nm wavelength range for water and lipid estimation. Specifically, a combination of 3 different wavelengths were sequentially selected in the 900-1000 nm range with 5 nm increments, and the condition numbers were calculated for the extinction coefficient matrices of the selected wavelength combinations. The optimal wavelength combination was determined by the smallest condition number, i.e., [920, 925, 1000] nm. We then processed the collected data and extracted water and lipid content using the 3 selected wavelengths

(i.e., 920, 925, and 1000 nm), and compared with the multi-wavelength approach (i.e., 900-1000 nm with 5 nm increments).

For the phantom study, the water and lipid concentrations extracted with the 3 wavelengths are shown in Table S2 below. While the 10%, 15%, and 20% lipid phantom groups had relatively small errors, the 5% lipid phantom group had particularly larger errors. Additionally, the average error for water content estimation calculated over all phantoms was 1.1±3.8% (versus 0.9±1.2% with 21 wavelengths), and the average error for lipid was -1.1±1.2% (versus -0.4±0.7% with 21 wavelengths). The results suggest that the use of 3 carefully selected wavelengths had overall larger errors in terms of both average and standard deviation values, with particularly large errors in the case of low lipid concentration (i.e., 5%).

For the lipid mapping of porcine tissue, the extracted absorption and reduced scattering maps at 920 nm are shown in Fig. S4 (b) and Fig. S4(c), respectively. The extracted lipid map with the 3 wavelengths is shown in Fig. S4(d). Compared with the lipid map extracted by 21 wavelengths (i.e., Fig. 5(d) in the main text), Fig. S4(d) appears noisier. This agrees with the phantom data above where the concentrations extracted by 3 wavelengths had larger errors.

The results of longitudinal monitoring of in vivo water content using 3 wavelengths (i.e., 920, 925, 1000 nm) are shown in Figure S5. As shown in Fig. S5(a) and (c), the extracted water maps appear noisier, which is similar to the results above in the lipid mapping of porcine tissue. In addition, as shown in Fig. S5(b), the results obtained by the 3 wavelengths were not able to differentiate the water content changes induced by injections of 0.1 ml versus 0.2 ml PBS.

Overall, the results above suggest that compared with the presented multiwavelength approach (i.e., 21 wavelengths in the 900-1000 nm range for water and lipid content extraction), the combination of 3 discrete wavelengths (i.e., [920, 925, 1000] nm) performs less well for different

applications including water and lipid content extraction on phantoms, *ex vivo* lipid mapping, and *in vivo* tissues water content monitoring.

3. Comparison of water and lipid content extraction with simulations

Following the procedures described for Fig. 4 of the main text, we calculated water and lipid extraction errors with different wavelength increments. Since the noise parameters of SWIR-MPI is unavailable, we assumed noise levels of 1%, 5%, and 10% respectively for the absorption values at different wavelengths. Since zero-mean Gaussian noise was used in the simulations, the magnitude of average percent errors was close to zero for different wavelength combinations (all below 0.04% for both water and lipid). Therefore, we used the standard deviation to evaluate the goodness of concentration extraction. Fig. S6 (a) shows the standard deviation of percent errors for water extraction using different wavelength increments under 1% Gaussian noise. The standard deviation of percent errors for the presented method, SWIR-MPI, and the case of only using 3 selected wavelengths (i.e., 920, 925, and 1000 nm) are shown by the blue curve, the red curve, and the black dashed line, respectively. Fig. S6 (b) shows the standard deviation of percent errors for lipid extraction under 1% Gaussian noise. Additionally, Fig. S6 (c) and (d) show the standard deviation of percent errors under 5% Gaussian noise; Fig. S6 (e) and (f) show the standard deviation of percent errors under 10% Gaussian noise. The results demonstrate that under different noise levels, the presented method has nearly identical performance on water content extraction compared to SWIR-MPI, while SWIR-MPI performs better for lipid content extraction. In contrast, the version of only using 3 selected wavelengths (i.e., 920, 925, and 1000 nm) consistently has the largest errors among the assessed methods.

Supplementary Note 3 – SNR analysis, comparison, and ways of improvement

It is also important to discuss about SNR and how to improve it. As an example, here we provide SNR analysis of the silicon-based camera used in this study versus the germanium camera used in SWIR-MPI. The quantum efficiency data of the silicon-based detector used in this study, and the germanium-based detector used in previous SWIR-MPI study are plotted in Fig. S7. In the 900-1000 nm wavelength range, the quantum efficiency of the silicon-based camera is 2.5-10.7%, whereas the quantum efficiency of the germanium-based camera is 28.9-37.2%. The average quantum efficiencies over the collected wavelengths in the 900-1000 nm range were 5.9% for the silicon-based camera and 34.9% for the germanium-based camera, respectively. Regarding SNR, it is determined by both signal and noise levels. Under the same illumination power (and collection optics, etc.), the difference in signal intensity would be dependent on the quantum efficiency. The noise part comes from the dark noise and readout noise of the camera. While the readout noise of the camera used in this study is not available in the data sheet, we conduct noise level analysis based the dark noise parameter. Specifically, the dark noise of the silicon-based camera and the germanium-based camera are 3.71 e-/s and 480 e-/s, respectively. The integration times utilized for the 900-1000 nm range with the silicon-based camera and the germanium-based camera were 50-130 ms and 2.3-8.4 ms, respectively. The average exposure times over the collected wavelengths in the 900-1000 nm range were 83.3 ms and 4.93 ms for the silicon-based camera and germanium-based camera, respectively. Under the same number of signal photons (e.g., 100), we calculate SNR as (average quantum efficiency × number of photons) / (dark noise × average integration time). Subsequently, the SNR for the silicon-based camera and the germanium-based camera are 19 and 15, respectively. The data shows that given those two specific detectors and integration times, the SNR of the silicon-based camera is moderately higher than that of the germanium-based camera. Furthermore, it is very important to note that the SNR could be

dramatically improved by utilizing cameras with higher quantum efficiency and lower noise. For example, more advanced silicon-based cameras (e.g., Zyla 5.5 sCMOS, Andor Technology) could have dark noise of 0.1 e-/s, which is over 37× lower than that of the FLIR camera used in this study. Similarly, some deep-cooled SWIR cameras (e.g., NIRvana LN, Princeton Instruments) could have dark noise of 10 e-/s (48× lower) and >60% quantum efficiency (nearly 2× higher) at 900-1000 nm wavelengths, leading to almost a hundred-fold improvement on SNR compared to the germanium-based camera.

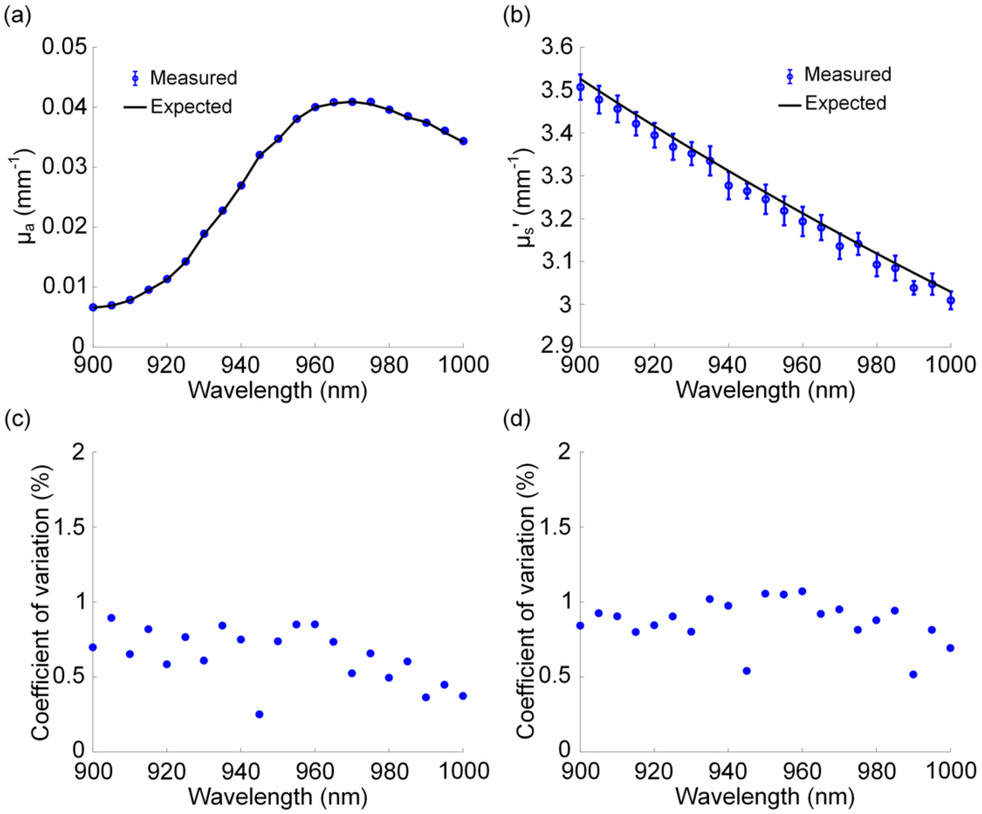

Fig. S1 (a) Error bar plot of the measured and expected optical absorption during the drift test. (b) Error bar plot of the measured and expected reduced scattering during the drift test. (c) Coefficient of variation of the optical absorption measurements during the drift test, with an average of 0.6±0.2%. (d) Coefficient of variation of the reduced scattering measurements during the drift test, with an average of 0.9±0.1%.

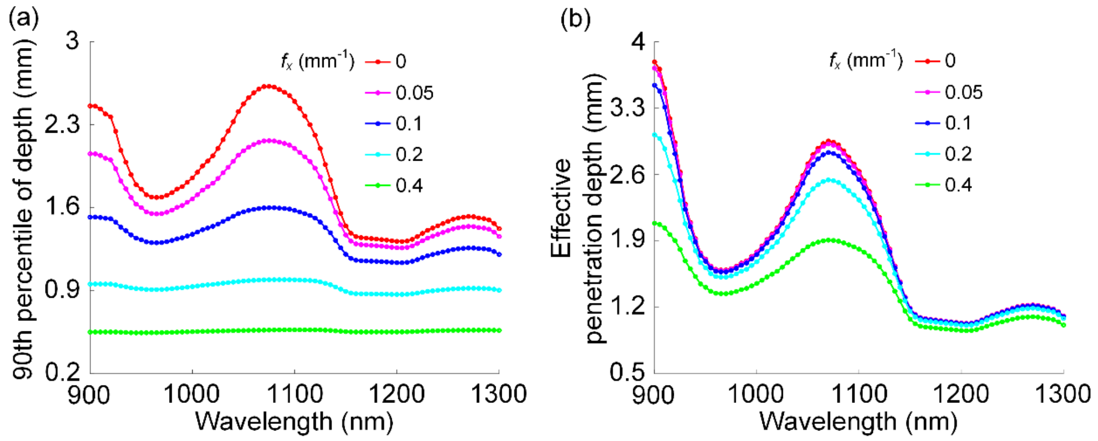

Fig. S2 (a) Photon 90th percentile depth penetration and (b) effective penetration depth of different spatial frequencies at 900-1300 nm wavelength range calculated with optical properties of tissue-mimicking 10% intralipid phantom.

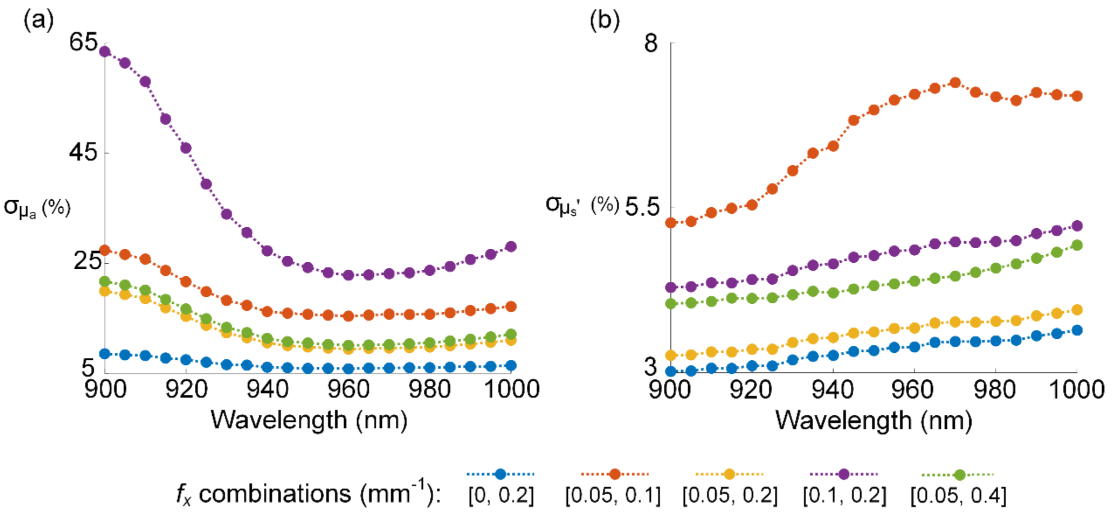

Fig. S3 Measurement uncertainties of (a) absorption and (b) reduced scattering with different spatial frequency pairs in the 900-1000 nm wavelength region.

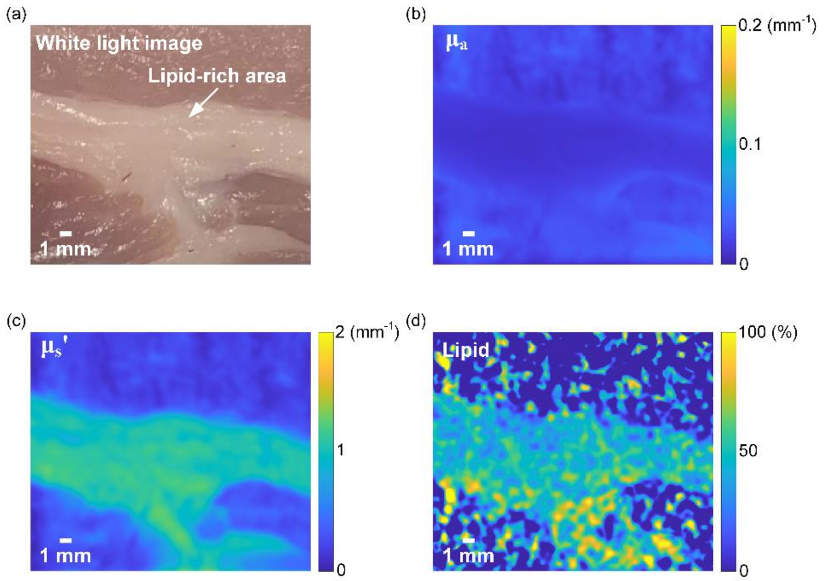

Fig. S4 (a) White light image of the porcine tissue. (b) Absorption map at 920 nm. (c) Reduced scattering map at 920 nm. (d) Lipid content map of the porcine tissue extracted using 3 wavelengths (i.e., 920, 925, 1000 nm).

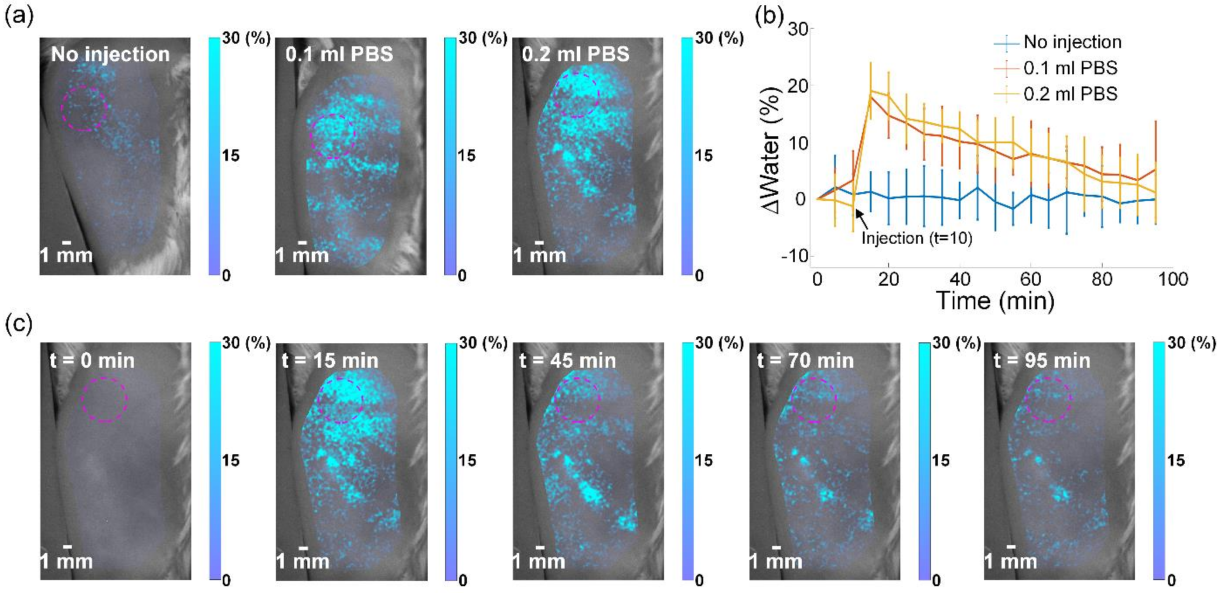

Fig. S5 Longitudinal monitoring of *in vivo* water content using 3 wavelengths (i.e., 920, 925, 1000 nm). (a) Representative mice are shown for the changes of water content immediately after subcutaneous injection of PBS (n = 4 per group). The magenta dashed circle is centered at the injection site and represents the 0.5 cm diameter region of interest used for calculating the average water content. (b) Time series of average water content changes for each group (error bars indicate standard deviations). (c) Temporal dynamics of water content on a representative mouse in the 0.2 ml group.

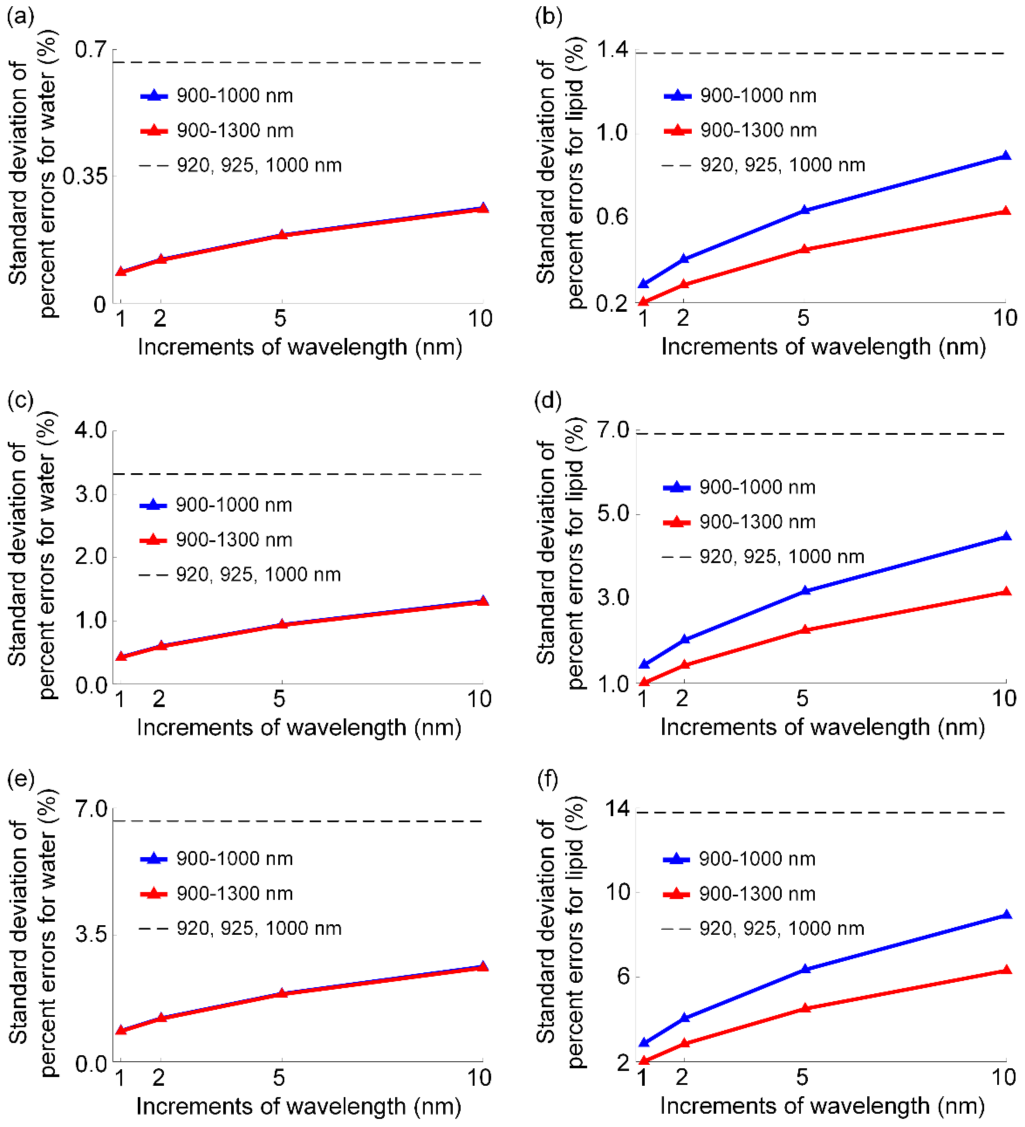

Fig. S6 Standard deviation of percent errors of extracted concentrations for water and lipid under different noise levels. (a) Water extraction with 1% Gaussian noise. (b) Lipid extraction with 1% Gaussian noise. (c) Water extraction with 5% Gaussian noise. (d) Lipid extraction with 5% Gaussian noise. (e) Water extraction with 10% Gaussian noise. (f) Lipid extraction with 10% Gaussian noise.

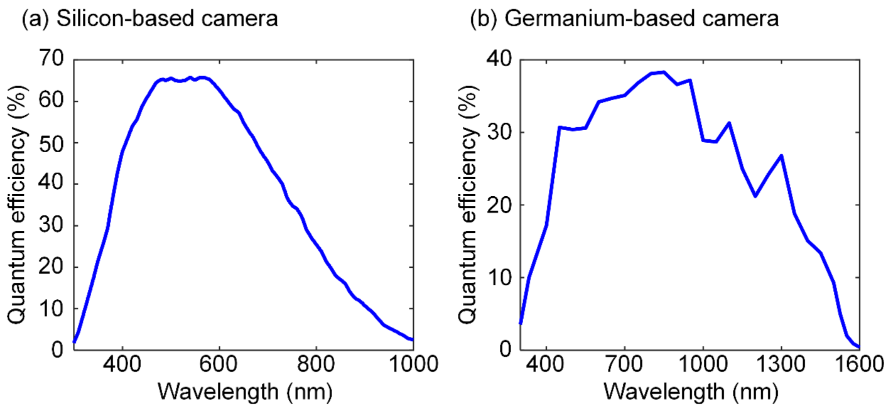

Fig. S7 Quantum efficiency of (a) the silicon-based camera used in this study and (b) the germanium-based camera used in SWIR-MPI.

Table S1 Accuracies of water and lipid concentration extraction in the drift test.

**Drift measurements**	**Known concentration**		**Extracted concentration**		**Error**	
	Water	Lipid	Water	Lipid	Water	Lipid
#1	90.0%	10.0%	89.8%	10.4%	-0.2%	0.4%
#2	90.0%	10.0%	89.9%	11.2%	-0.1%	1.2%
#3	90.0%	10.0%	91.0%	10.8%	1.0%	0.8%
#4	90.0%	10.0%	90.5%	10.2%	0.5%	0.2%
#5	90.0%	10.0%	90.0%	10.1%	0.0%	0.1%
**Average (± standard deviation)**	90.0%	10.0%	90.2% (±0.5%)	10.5% (±0.5%)	0.2% (±0.5%)	0.5% (±0.5%)

Table S2 Extracted water and lipid concentrations with 3 wavelengths (i.e., 920, 925, and 1000 nm). Five phantoms were measured per water-lipid recipe.

**Known concentrations**		**Extracted concentrations**		**Errors**	
Water	Lipid	Water	Lipid	Water	Lipid
95.0%	5.0%	102.3±0.4%	2.4±1.3%	7.3±0.4%	-2.6±1.3%
90.0%	10.0%	89.8±0.2%	9.9±0.4%	-0.2±0.2%	-0.1±0.4%
85.0%	15.0%	83.0±0.9%	14.4±0.3%	-2.0±0.9%	-0.6±0.3%
80.0%	20.0%	79.5±0.3%	19.0±0.8%	-0.5±0.3%	-1.0±0.8%
